# Novel Mangosteen-Leaves-Based Marker Ink: Color Lightness, Viscosity, Optimized Composition, and Microstructural Analysis

**DOI:** 10.3390/polym13101581

**Published:** 2021-05-14

**Authors:** Mohd Salahuddin Mohd Basri, Brenda Liew Min Ren, Rosnita A. Talib, Rabitah Zakaria, Siti Hasnah Kamarudin

**Affiliations:** 1 Department of Process and Food Engineering, Faculty of Engineering, Universiti Putra Malaysia (UPM), Serdang 43400, Selangor, Malaysia; brendaliew96@gmail.com (B.L.M.R.); rosnita@upm.edu.my (R.A.T.); rabitah@upm.edu.my (R.Z.); 2 Laboratory of Halal Science Research, Halal Products Research Institute, Universiti Putra Malaysia (UPM), Serdang 43400, Selangor, Malaysia; 3 Laboratory of Biopolymer and Derivatives, Institute of Tropical Forestry and Forest Products (INTROP), Universiti Putra Malaysia (UPM), Serdang 43400, Selangor, Malaysia; 4 School of Industrial Technology, Faculty of Applied Sciences, Universiti Teknologi MARA (UiTM), Shah Alam 40450, Selangor, Malaysia; sitihasnahkam@uitm.edu.my

**Keywords:** mangosteen leaves, marker ink, color lightness, viscosity, optimization, RSM

## Abstract

Dry mangosteen leaves are one of the raw materials used to produce marker ink. However, research using this free and abundant resource is rather limited. The less efficient one-factor-at-a-time (OFAT) approach was mostly used in past studies on plant-based marker ink. The use of statistical analysis and the regression coefficient model (mathematical model) was considered essential in predicting the best combination of factors in formulating mangosteen leaf-based marker ink. Ideally, ink should have maximum color lightness, minimum viscosity, and fast-drying speed. The objective of this study to study the effect of glycerol and carboxymethyl cellulose (CMC) on the color lightness and viscosity of mangosteen-leaves-based marker ink. The viscosity, color lightness, and drying properties of the ink were tested, the significant effect of glycerol and CMC (responses) on ink properties was identified and the prediction model on the optimum value of the responses was developed by using response surface methodology (RSM). The microstructure of mangosteen leaves was analyzed to study the surface morphology and cell structure during dye extraction. A low amount of glycerol used was found to increase the value of color lightness. A decrease in CMC amounts resulted in low viscosity of marker ink. The optimum formulation for the ink can be achieved when the weight percents of glycerol, benzalkonium chloride, ferrous sulphate, and CMC are set at 5, 5, 1, and 3, respectively. SEM micrographs showed the greatest amount of cell wall structure collapse on samples boiled with the lowest amount of glycerol.

## 1. Introduction

For centuries, ink is made from natural products such as berries, barks, and extracts of leaves. These have been used as raw materials to create various colors and to produce ink, dye, or paint when mixed with other substances. Early records showed that tea leaves had been used to make ink due to their shades that able to produce yellow, green, brown, or black ink [1]. Ink has been studied in a wide area of applications including inkjet printing [2,3] and bio-ink [4]. Inks are commonly made today from non-renewable synthetic resources such as petroleum- and chemical-based solvents, which are harmful to both users and the environment. Unprotected contact with ink may cause severe headaches, skin irritation, or damage to the nervous system likely due to the effects of solvents or pigment, such as p-anisidine, in the ink.

Noah et al. [5] conducted a preliminary study on the production of brown ink from *Gmelina arborea* fruit extract. The objective was to assess the potential of producing brown ink from the extract and compare the ink produced with commercial inks using an ink flotation test. Five different concentration levels (100%, 80%, 60%, 40%, and 20%) of ink were produced using ethanol as diluent and coconut vinegar as an additive to preserve the ink from biodegradation and to enhance its stability and permanence on paper once dried. The results showed that the production of brown ink from *Gmelina arborea* fruit extract was feasible.

Nwafulugo et al. [6] studied the marker ink production from a huckleberry (*Vaccinium membranaceum*) ink extract. The liquid was extracted by cooking and mashing the berries with the addition of apple cider (vinegar) as a solvent extractor. The extracted liquid was then mixed with 108 mL of gum arabic, 18 mL of methanol, and two teaspoons of dye, yielding an ink sample in each mixture. The physical properties of the sample were tested and compared to commercial ink. The results showed that the ink produced was of high quality, with a pH of 9.3, drying time of 2.3 s, and viscosity of 9.5 × 10^–4^ Ns/m^2^.

Dagde et al. [7] studied the formulation of whiteboard marker ink using locally sourced raw materials such as natural wood from mango (*Mangifera* sp.), rubber (*Hevea brasiliensis*), and ugba or oil bean trees (*Pentaclethra macrophylla*). The pyrolysis process carried out on the different woods produced the final product called charcoal. In terms of odor, color, hazardous reaction, pH, density, and viscosity, the various ink samples were formulated and compared to international standards. The results showed a good match, indicating that the whiteboard marker ink produced is reliable and of high quality. The pH results for ugba (oil bean), rubber, and mango charcoal ink are 5.43, 6.79, and 7.41, respectively. Ugba (oil bean) charcoal ink showed the highest viscosity followed by rubber and mango charcoal ink.

Mangosteen (*Garcinia mangostana*) is planted in most of the countries of Southeast Asia—including Indonesia, Malaysia, the Philippines, and Thailand. Mature mangosteen trees can range from 6 to 25 m in height. The primary active components of the fruits are known as xanthones, which have several benefits, including anti-inflammatory properties, anti-allergic, and anti-convulsants [8]. The rind is reported to contain from 7% to 15% tannin and is used for tanning leather and dyeing in black color [9]. The rind of the mangosteen is also known for use as an ingredient in soap, shampoo, and conditioner [10].

One of the most important uses of mangosteen is as a source of natural dye for fabric and solar cells because of its prominent color. Fully ripened mangosteen pericarp contains anthocyanins, such as cyanidin-3-sophoroside and cyanidin-3-glucoside, which contribute to the dark purple or red coloration of its pericarp [11]. Anthocyanin can be used as a natural color extract to replace synthetic colorants because of its light color and higher solubility. Food scientists and horticulturists have meticulously examined anthocyanins because of their strong effect on the coloring quality of pre-harvested and post-harvested vegetables and fruits [12]. Anthocyanins are grouped under the family of flavonoid compounds, which contain solvable water pigments. These color pigments are also present in fruits and flowers and able to attract insects and animals [13].

Other dye compounds, such as tannin, can also be extracted from mangosteen for its brown coloring property [14]. These natural dyes exhibit excellent potential in the textile industry. They can be obtained easily from the leaves, pericarp fruit waste, and barks of the mangosteen tree. Due to their biodegradable and nontoxic properties, they are safe to the environment compared with synthetic dyes. Researchers for instance have successfully dyed cotton fabrics using mangosteen extract. In general, mangosteen sources have been used for a variety of purposes, ranging from industrial products to advanced technology and biomedical applications. The use of its fruits has recently been studied in postharvest biology, food science, and engineering fields.

Carboxymethyl cellulose (CMC) and glycerol are the additives used in the production of marker ink. CMC, also known as cellulose gum is a cellulose derivative with carboxymethyl groups (–CH_2_–COOH) incorporated into some of the hydroxyl groups of the glucopyranose monomers that contribute to the cellulose backbone. It is often used as its sodium salt, sodium CMC. Habib and Khoda [15] studied the development of clay-based novel bio-ink by optimizing the composition of materials, which are montmorillonite (MMT), CMC, and sodium alginate. CMC is a water-soluble polysaccharide with a high molecular weight that is used as a viscosity modifier or thickener. It is reported that the binding of CMC’s matrix protein assisted in cell migration and cell attachment. The incremental addition of CMC into alginate and MMT mixture makes the bio-ink more viscous. Bio-ink with very high viscosity requires high air pressure to suspend the filament that may limit the use of available bio-printer. The rheological properties of CMC were investigated by Ghannam and Esmail [16]. An increase in CMC concentration in the solutions changes the behavior from Newtonian to shear thinning. The higher the concentration, the lower the flow behavior index. The increase in CMC concentration is accompanied by stronger time-dependence of the rheological properties.

Glycerol (1,2,3-propanetriol) is a colorless, odorless, viscous liquid with a sweet taste. It is made from both natural and petrochemical substances [17]. Presently, the quantity of glycerol accounted for approximately 160,000 tonnes annually and is used for technical applications; this is expected to grow at an annual rate of 2.8%. Glycerol is typically used as a softener and plasticizer in alkyd resins and regenerated cellulose to determine properties in surface coatings and paints including flexibility, pliability, and toughness. It serves as binders in paints and inks [18]. Medeiros et al. [19] investigated the effects of glycerol concentration on thermophysical properties of the water-glycerol solution. The results showed that with an increase in the amount of glycerol, the density and dynamic viscosity were increased.

Although many studies on marker ink have been conducted based on plant material, research on the development and production of marker ink using dried mangosteen leaves is rather limited. The number of studies looking into the effects of various factors on the properties of marker ink is still minimal. Besides, the proper method in determining the optimum formulation using statistical optimization has not been applied in these studies.

Despite a large number of publications on biodegradable and environmentally friendly marker inks reported in the literature in recent years [7,20,21,22], most studies were carried out in the one-factor-a-time (OFAT) approach. For greater efficiency, the application of statistical analysis and regression coefficient (mathematical model) are necessary for better prediction on the optimum composition of marker ink with high color lightness and low viscosity. Compared to OFAT, the design of the experiment (DOE) provides many advantages, including low requirements for resources (experimental runs, time, material, and manpower), accurate measurement of main effects and interactions, and the ability to simultaneously analyze several variables [23]. Furthermore, the response surface methodology (RSM), which was originally coined by Box and Wilson [24], is used commonly as a mathematical model for enquiring into significant effects, interactions, and optimization studies. The central composite design (CCD) has been proven to be the best model for analysis [25].

Since the RSM approach, specifically CCD, has been widely used in polymer optimization [26,27,28,29], it will, therefore, be adopted in this study. The main objectives of this study are: (i) to identify the significant effects of different additives on the viscosity, color lightness, and drying speed of mangosteen-leaves-based marker ink; (ii) to determine the optimum composition of the mangosteen-leaves-based marker ink using RSM; and (iii) to study the microstructure of mangosteen leaves during the dye extraction process.

## 2. Materials and Methods

### 2.1. Factors and Levels of the Design of Experiment

In the study, CMC and glycerol—designated as V_1_ and V_2_ respectively—were chosen as factors. Other factors, such as ferrous sulphate and benzalkonium chloride, were kept constant at 1 and 5 wt %, respectively. Five weight percent (5 wt %) denoted as 5% of the total weight of the leave’s boiled water. Factors and levels used in the DOE are shown in Table 1.

### 2.2. Design of Experiment

RSM was used to design and conduct experiments due to its lower experimental runs as compared to the conventional experiment. Table 2 shows the difference in the total number of experimental runs between full-design RSM and a full factorial design (classical method) based on five-level factors. The result shows that for analyzing four factors, the full-design RSM only requires a minimum of 30 experimental runs (with one replication) as compared to 625 for the full factorial design.

At each design stage, five levels and two factors were applied in the CCD and with three replications for a total of 39 experimental runs. The factors were selected based on preliminary lab work, their significant effect on the responses, and working range (workability). Table 3 displays the complete CCD with coded and uncoded levels of these factors. The value for the total block is 1 with the experiments carried out in randomized order. For analysis using RSM, V_1_ = V_2_ = 0 represent the center point. Replicating an entire DOE to increase the number of data points can be expensive and time-consuming. An alternative way to increase the accuracy of the analysis is to use center points. By increasing the number of center points in the DOE, the probability of analyzing data and perform prediction of the optimum value for the factors can be increased. Therefore, a total of 15 experimental runs has been designed for center points resulting in a total of 39 experimental runs.

The significance of the main factors and their interactions is calculated using the analysis of variance (ANOVA). The value of 95% was set as the significance level which reflected the *p*-value of 0.05. Based on the value of the correlation coefficient (R^2^), the regression coefficient model (mathematical model) developed in the ANOVA table is used for optimization purposes. Experimental data are fitted with a second-order polynomial model to obtain the regression coefficient model. The general mathematical model obtained from the analysis is shown in Equation (1)

(1) $$ϒ=\beta_{0}+\sum_{t=1}^{3}\beta_{i}X_{i}+\sum_{i}^{3}\beta_{ii}X_{i}^{2}+\sum_{i-1}^{2}\sum_{j=i+1}^{3}\beta_{ij}X_{i}X_{j}$$

where Y is the response, *β*_0_, *β_i_*, *β_ii_*, and *β_ij_* are regression coefficients for the intercept, linear, quadratic, and interaction terms, respectively. *X_i_* and *X_j_* are coded values for the independent variables [30].

The desirability function was used to optimize the central composite design. The desirability function was created in this study to minimize the viscosity and color lightness of the ink. Validation was carried out by triplicate under optimal conditions.

### 2.3. Raw Materials and Sample Preparation

**Figure 1:**
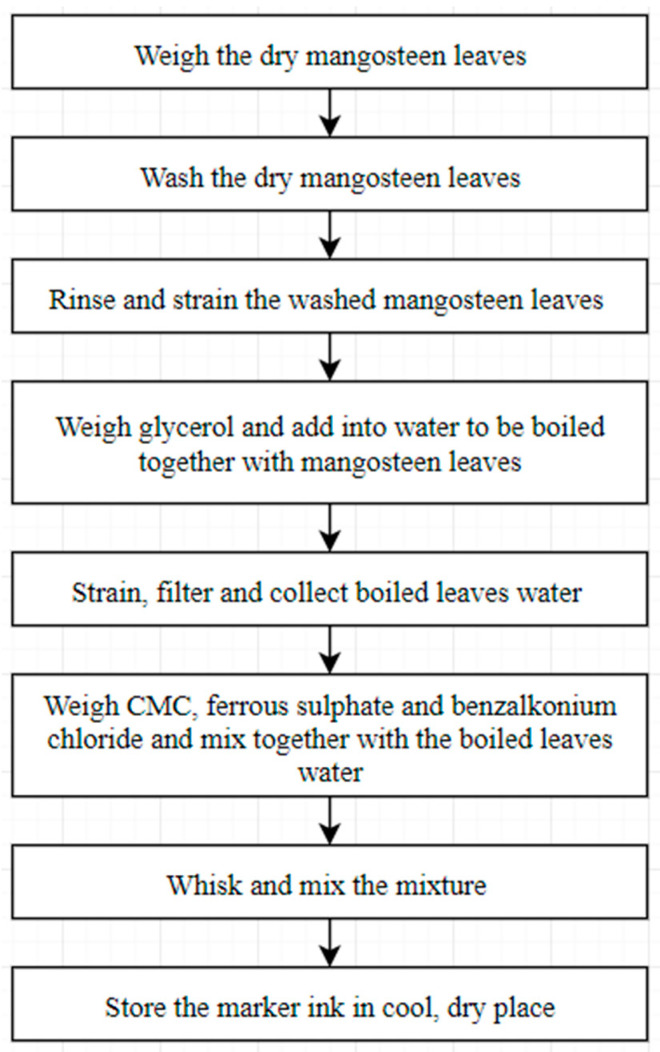
Fabrication process of mangosteen-leaves-based marker ink.

Dry mangosteen leaves, used as the main component in the marker ink production, were obtained from the Family Fruits Farm, Batang Kali, Selangor, Malaysia. The CMC, ferrous sulphate, benzalkonium chloride, and glycerol used in this study was purchased from Merck KGaA, Darmstadt, Germany. The specification for glycerol is colorless, and it contains 1.0% water, viscous, and clear liquid. CMC is an odorless white powder with a viscosity of 400 to 800 centipoise (cp), in a 2% solution in water at 25 °C. Ferrous sulphate has a density of 1.898 g/mL at 25 °C while benzalkonium chloride is a clear, colorless liquid with a concentration of 50 to 55 *w*/*w*%. The marker ink was prepared by following the steps, as shown in Figure 1.

Approximately 75 g of dry mangosteen leaves were first weighed by using a digital weighing scale (Mettler Toledo, Columbus, OH, USA) which was able to read up to 0.001 g and for a sample weight up to 220 g. These were then washed thoroughly and rinsed. Glycerol ranging from 15 g to 62.5 g was weighed by using a digital weighing scale and 375 mL of water was measured using a measuring cylinder, and both were mixed in a beaker and thoroughly stirred for 30 s. The mixture was boiled together on a hot plate (Thermo Fisher Scientific, Waltham, MA, USA) at a heating degree of 8 (circa. 300 °C) for 30 min to increase the extraction rate of dye from the leaves. The hot plate can be heated up to 350 °C and the heating rate can be set from 0% to 100% at 1% intervals. The beaker was covered with plastic foil, to reduce vapor evaporation. Once boiled, the heating degree was reduced to 2 (circa. 100 °C). After boiling the hot plate was switched off, and the boiled mixture was collected into a container using a strainer.

CMC, ferrous sulphate, and benzalkonium chloride of 1–12.5 g, 3 g, and 15 g, respectively were weighed using a digital scale and added into the boiled mixture containing mangosteen leaves. To avoid clumping, the mixture was thoroughly stirred with a whisker, resulting in a smooth and homogeneous ink solution. The ink produced was stored in a glass container, sealed with an airtight cover, and kept in a cool, dry place for 24 h prior to testing.

### 2.4. Viscosity Test

The ink viscosity was investigated to develop a low viscous ink solution with a high drying rate. Ink samples were accordingly tested 24 h following preparation. The advanced AR-G2 rheometer (TA Instruments, New Castle, DE, USA) was used to conduct the viscosity test in accordance with the ASTM D-445 standard at 23 °C. A cone plate geometry with properties of 60 mm cone diameter, 1:0:0 cone angle ratio (deg:min:s), and 30 μm truncation was installed to the rheometer. The zero-gap was set for 30 µm and 5 g ink samples were placed on the Peltier plate by using a dropper. The experiment was initiated by clicking the ‘run’ icon, and the results were recorded.

### 2.5. Color Lightness Test

Color lightness was evaluated using a HunterLAB Ultrascan PRO d/8 Spectrophotometer (Hunter Associates Laboratory, Inc., Reston, VA, USA) operated in accordance with ASTM E1348. Approximately 50 mL of ink sample was used and placed in front of the porthole and the readings recorded. The colorimetric values were reported in terms of CIE color analysis for lightness (L*). The lightness, L*, represents the darkest black at L* = 0, and the brightest white at L* = 100.

### 2.6. Thermogravimetric Analysis

Thermogravimetric analysis (TGA) was conducted on marker ink samples using Mettler Toledo AG-TGA/SDTA (Model 851e, Mettler Toledo AG, Greifensee, Zürich, Switzerland). For specimen preparation, 20 mg of ink was used for each test. Nitrogen was used as a medium and the specimens were heated from 20 °C to 200 °C with a rate of 25 °C/min. Three ink samples that exhibited high, moderate, and low color lightness were tested to observe variation in their drying rates. As drying progressed, the weight of coating accordingly decreased due to solvent evaporation. The amount of solvent loss with time was monitored through mass change as recorded on the balance [31].

The drying rate of coating is the weight of solvent loss per time divided by the area of evaporation and this can be calculated using Equation (2) where R_mass_ is the evaporation rate, W is the weight of sample at a specific time, t is the time, ∆t is the time interval between measurements, and A is the evaporation area.

(2) $$R_{mass}=-\frac{dW}{Adt}\approx -\frac{W_{t+\Delta t}-W_{t}}{A\Delta t}$$

### 2.7. Microstructure of Rice Husk Ash

The scanning electron microscope (SEM) is one of the most versatile instruments available for the examination and analysis of microstructure morphology of plant materials [32]. Since the discovery that electrons can be deflected by a magnet, electron microscopy was developed by substituting the light source with a high-energy electron beam [33].

SEM analysis was carried out using the Hitachi S-3400N variable SEM. The microstructure images of mangosteen leaves, including raw samples, or those boiled without glycerol and with glycerol, and samples which exhibited high, moderate, and low color lightness and viscosity properties. Samples were prepared by placing on aluminum sample stubs with carbon conductive tape and were then sputter-coated with gold-palladium using Baltec SCD-005 Sputter Coater to produce high quality and bright image. The stub with coated samples was inserted into the sample chamber of the SEM for viewing.

## 3. Results and Discussion

The complete design matrix and response values of viscosity and color lightness are given in Table 4. Before designing an experiment using RSM, a preliminary study or screening process using a fractional factorial design (FrFD) method is required. The screening process is essential to identify the most significant factors and to reduce the number of less significant ones when designing an experiment using RSM. For the screening process, four factors including glycerol, ferrous sulphate, benzalkonium chloride, and CMC were studied. Glycerol and CMC showed the most significant effect on the responses and therefore were selected as factors in this study. Data were analyzed using MINITAB.

### 3.1. Statistical Analysis of Color Lightness and Viscosity Properties

A linear regression model was fitted to the experimental data using the least square technique. Several main parameters were considered in evaluating the statistical results, namely the coefficients of regression, the standard error of coefficient, and the *p*-value of each factor and its interactions for both responses which are color lightness and viscosity. The results in Table 5 indicated that all factors and interaction effects were highly significant (*p* < 0.000) except for V_2_ with *p* < 0.113 and ‘V_2_* V_2_′ with *p* < 0.529. Values for R^2^ = 0.9873 and R^2^ (adjusted) = 0.9858 were considerably high, which indicated that 98.73% of sample variation in the response was attributed to the factors.

For viscosity, the *p*-value for both factors (color lightness and viscosity) and their interactions were considered significant at below the confidence level of 95% (*p* of 0.050). The results shown in Table 6 indicated that all factors and interaction effects were significant. The *p* values of all factors and their interactions were highly significant (*p* < 0.000) except for V_1_ which did not affect the viscosity of the ink (*p* < 0.382). Values for R^2^ = 0.9292 and R^2^ (adjusted) = 0.9209 were considered very high, which indicated that 92.92% of the sample variation in the response was attributed to the independent variables.

Equations (3) and (4) represent the regression models for the color lightness and viscosity, respectively.

(3) $${ϒ}_{COL}=25.7739+1.0331\left(V_{1}\right)+0.0347\left(V_{2}\right)+0.2607\left({V_{1}}^{2}\right)+0.0098\left({V_{2}}^{2}\right)$$

(4) $${ϒ}_{VIS}=0.0938-0.0073\left(V_{1}\right)+0.1431\left(V_{2}\right)-0.0235\left({V_{1}}^{2}\right)-0.0593\left({V_{2}}^{2}\right)$$

where Y*_COL_* and Y*_VIS_* represent the responses which are color lightness and viscosity, respectively whereas *V*_1_ and *V*_2_ are the decoded values of glycerol and CMC, respectively. The regression models can be used to calculate and analyze the effect of factors on the properties of plant-based marker ink.

### 3.2. Effect of Factors on Color Lightness and Viscosity

**Figure 2:**
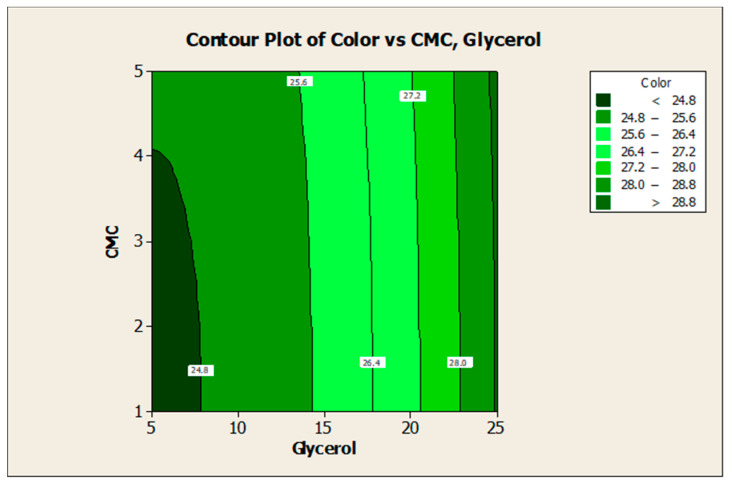
Contour plot for the effect of glycerol and CMC concentration on the color lightness of mangosteen-leaves-based marker ink.

**Figure 3:**
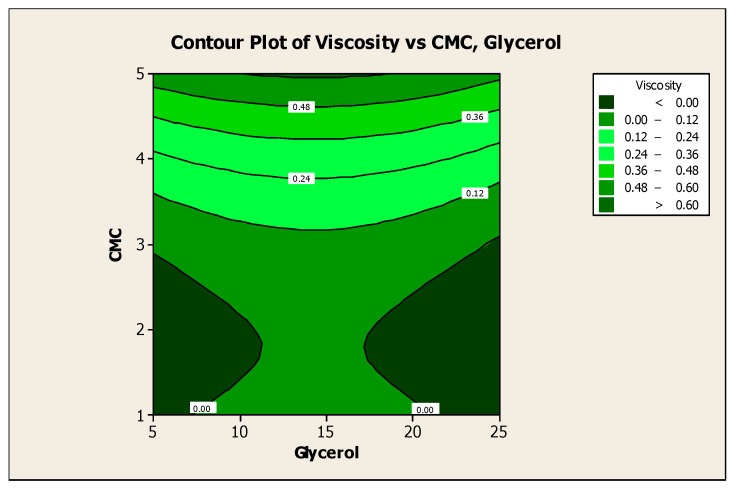
Contour plot for the effect of glycerol and CMC concentration on the viscosity of mangosteen-leaves-based marker ink.

ANOVA and regression models were used to analyze the effect of various factors on the properties of the plant-based marker ink. Contour plots were used for better illustration. Figure 2 and Figure 3 illustrate the effect of glycerol (V_1_) and CMC (V_2_) on the responses, respectively. The findings show that higher V_1_ resulted in higher color lightness, and lower V_2_ resulted in lower viscosity. Based on Figure 2, the color lightness of mangosteen-leaves-based marker ink decreased when the ratio of glycerol concentration in the ink formulation is approaching 5. This indicates that a low concentration of glycerol reduces the value of color lightness in the ink.

An increase in the amount of glycerol in a liquid may increase the boiling temperature of the liquid [19]. Glycerol is alcohol with low vapor pressure. It changes its colligative properties when mixed with water, increasing the boiling point and lowering the melting point of the solution. According to Kowalska et al. [34], an increase in boiling temperature from 20 to 80 °C (glycerol content of 30% and below) may consequently decrease the total dye extraction yield. The study also discovered that increasing the glycerol concentration from 30% to 80%, while maintaining the same boiling temperature reduced the amount of extracted anthocyanins, which is in conformance with the results obtained in this study. Anthocyanin pigments are known as natural colorants due to their wide range of colors and high solubility in aqueous media [35].

The contour plot in Figure 3 shows the effect of glycerol and CMC concentration on the viscosity of plant-based marker ink. At a glycerol concentration of 5, the viscosity decreases as the CMC concentration decreases. Ghannam and Esmail [16] investigated the rheological properties of CMC solutions in a concentration ranging from 1% to 5%. When the concentration was lowest, they observed Newtonian behavior, while at higher concentrations, they observed non-Newtonian behavior such as pseudoplastic, thixotropic, and viscoelastic behavior.

Similar results were obtained by Edali et al. [36], who investigated the rheological behavior of CMC solutions also at higher concentrations. They found that the solution possessed both non-Newtonian and viscoelastic properties. Other studies also concluded that the apparent viscosity of CMC solution increased with rising concentrations. The results were obtained from the flow curves of the CMC solutions at different concentrations plotted over a log–log scale. This rise in apparent viscosity is due to the increase in the interactions between the CMC molecules [37].

### 3.3. Optimization of the Responses

**Figure 4:**
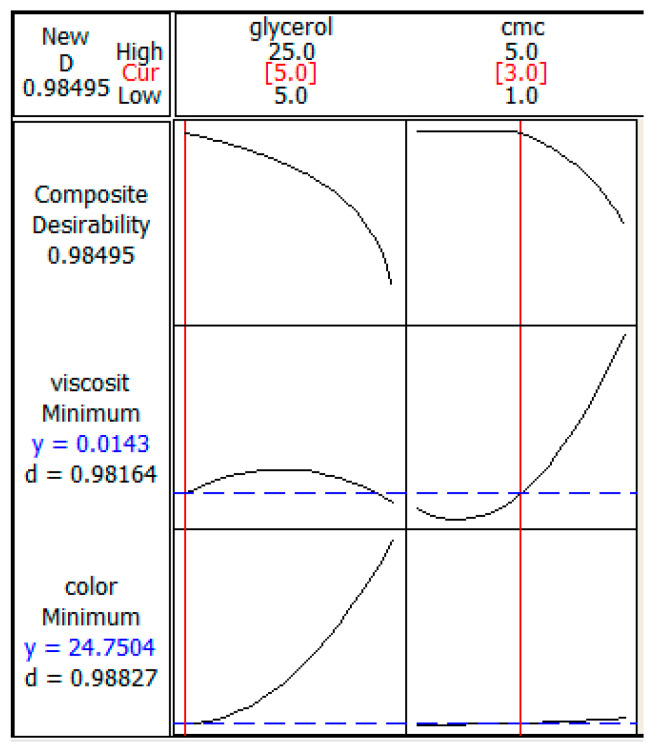
Optimization plot for glycerol and CMC.

The optimum formulation of marker ink can be achieved with the combination of glycerol ratio = 5 and CMC ratio = 3, as shown in Figure 4. The desirability of optimization was calculated as 0.98495, indicating that all parameters were within the target, which was to obtain desirable ink properties.

The value of composite desirability was calculated at 0.98495, which is high and which proved that the prediction is accurate. It also indicates that all parameters were within the target of obtaining the desirable ink properties. The optimization was completed within a set of parameters. For color properties, the target and upper values were set at 24.7 and 29.0, respectively. The target and upper boundary values for viscosity properties were set at 0.001 and 0.7278, respectively.

### 3.4. Experimental Validation

From Table 7, it is clear that the average error for color lightness and viscosity were well below 15% at only 0.50% and 9.09%, respectively. It was concluded that the regression model established using this method was able to optimize accurately the values for the responses.

### 3.5. Microstructure Analysis of Mangosteen Leaves

Five samples were selected to determine the microstructure of mangosteen leaves and the drying properties of the marker ink. The samples were dried mangosteen leaves, boiled leaves without glycerol, and with glycerol showing low (sample S26), moderate (sample S19), and high (sample S35) value of color lightness. The sample was analyzed using an SEM.

**Figure 5:**
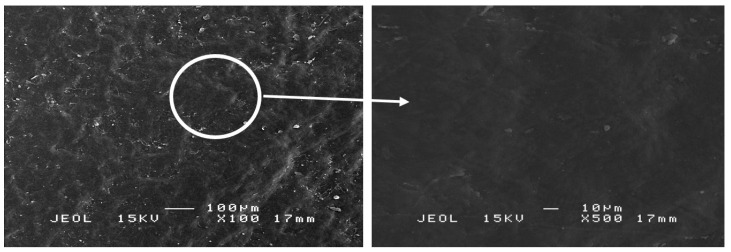
SEM micrographs of dry mangosteen leaves at 100× (**left**) and 500× (**right**) magnification.

Figure 5 illustrates SEM micrographs of dry mangosteen leaves at 100× and 500× magnifications. The former magnification shows an image with a relatively corrugated surface, in the form of wrinkles, developed on the leaf samples. The degree of surface corrugation was attributed to the drying process of the leaves at ambient temperature. The cell structure shrank slightly as a result of water evaporating from the leaves. The latter magnification shows no pores on the smooth surface of the dry leaves. This confirms that the leaves dried due to the evaporation of water at ambient temperature and not because of the influence of elevated temperature. In this stage, the drying shrinkage was closely related to the total volume of evaporated water [38].

Glycerol was found to exert the most significant effect on color lightness as compared to that of other additives. SEM scanning was conducted to investigate the effect of different amounts of glycerol on the microstructure of mangosteen leaves. Its addition will subsequently affect the amount of dye extracted from the leaves.

**Figure 6:**
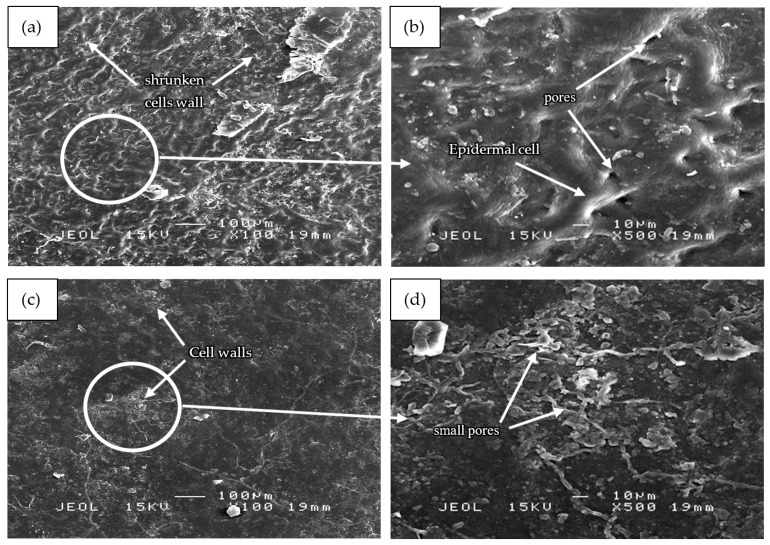

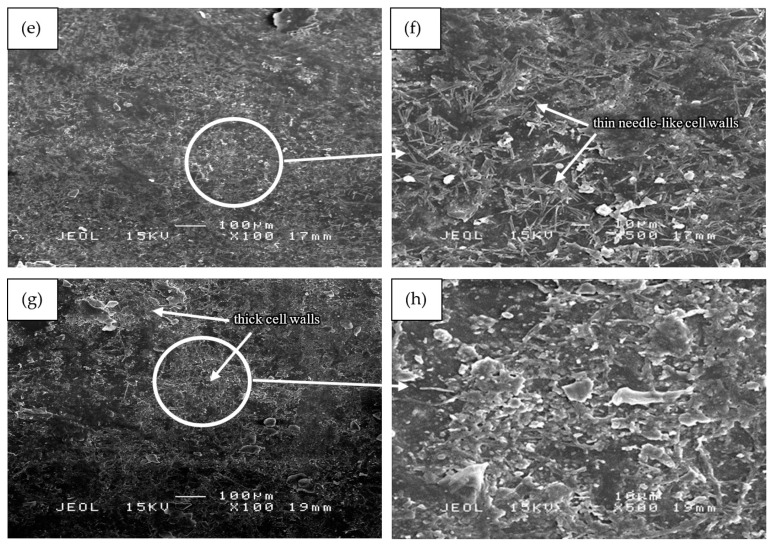
SEM micrographs of (**a**,**b**) boiled dry mangosteen leaves without glycerol, (**c**,**d**) sample S35, (**e**,**f**) sample S19, and (**g**,**h**) sample S26 at 100× and 500× magnification.

Based on Figure 6, the SEM micrographs of all samples showed corrugated leaf surface attributed to cell shrinkage. Figure 6a shows shrunken cells but with most of the cell wall structures remaining intact adjoining each other. Under high-temperature conditions, the leaves developed a folded cuticle on the adaxial epidermis layer [39]. Further magnification at 500× revealed a more definite structure of the leaf samples as compared to those under 100× magnification. Based on Figure 6b, boiled mangosteen leaves without glycerol showed good bonding between cell wall structures and considerably smooth surfaces. The dye was extracted from the leaves by passing it through the small pores between the cells. These pores were observed at the intercellular space, between neighboring epidermal cells. The moderate and large size of irregular oblong pores and disrupted orientation of membranes were formed due to heat stress during the boiling process.

A few recent studies described the effect of high temperature on the anatomical and ultrastructural changes which occurred in the leaf preparations including its organization, the protective outer layer of epidermis and cuticle as well as stomata [40]. The result is in agreement with that of Salem-Fnayou, Bouamama, Ghorbel and Mliki [39] which proved that a high-temperature condition can result in the folded cuticle and disrupted cell wall organization on the adaxial epidermis layer.

The microstructures of Figure 6a,c were found to be quite similar since the cell wall structures were still intact and adjoining each other. However, it can be seen that cell wall structures in sample S35 in Figure 6c,d had collapsed in certain areas and small pores had developed. The occurrence was due to the highest amount of glycerol used in boiling the sample. At high concentrations, glycerol is capable of altering plant cell structure. However, the collapse of cell wall structures is not as severe as samples S19 and S26. Glycerol is generally used as a solvent in water to extract natural pigments. The alcohol also acts as a pre-treatment agent and affects the final color produced through improving color performance [41].

Figure 6e of sample S19 covered with a thin needle-like and small-sized collapsed cell wall structures which mostly covered the leaf surface. Sample S26, as seen in Figure 6g, showed the highest amount of collapsed cell wall when the least amount of glycerol was used in boiling. The sample, therefore, showed the most significant effect of glycerol and in agreement with Figure 2. The thicker cell wall structures in sample S26 as compared to that of sample S19 can be visualized based on the brightness of the aggregated structures. The formation of the large and thick cell wall structures was probably due to the agglomeration of the high amount of the collapsed small-sized cell wall structures. Due to the large surface area and strong attractive interaction between particles, the aggregation process must have occurred as similarly recorded by other researchers [42]. Aggregation caused the fracture mechanism to change, with the fracture initiating and developing from the aggregation center [43].

The findings suggest that a decrease in the amount of glycerol in boiled water may alter the cell wall structure of the leaves and subsequently increase the dye extracted. The role of glycerol as dye extraction solvent has two main effects: first, the weakening of the membrane structures of the cells in which the anthocyanin are stored in cell vacuoles, and second, the transformation of the extracted anthocyanin into their flavylium form, which is the most stable form [44]. This condition has been extensively used in technological processes to extract dye from other natural sources such as fresh berries and grape pomace [45].

### 3.6. Drying Properties of Marker Ink

**Figure 7:**
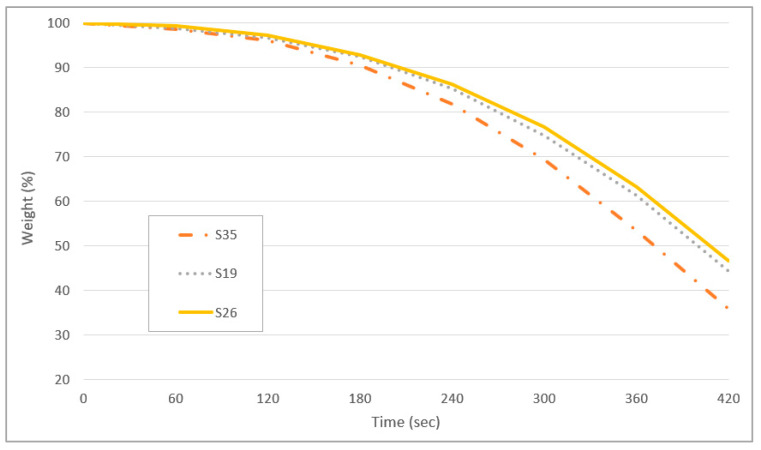
Weight loss as a function of time of sample S35, S19, and S26.

The thermogravimetric analysis (TGA) is a suitable alternative method in determining the drying rate of a solution as measured through sample weight loss during a drying process [31]. The drying rate is affected by the viscosity of a fluid and the rate of evaporation of a solvent [46]. In this study, four ink samples tested exhibited low, moderate, high, and optimum color lightness and viscosity properties. To better visualize the weight loss of the ink samples during the TGA test, a graph of weight loss (in%) as a function of time was plotted as shown in Figure 7.

The weight loss for all samples for the first 120 s was very minimal, with a maximum of approximately 4% loss recorded for sample S35. As the test temperature was increased the rate of 25 °C for every 60 s, the initial drying rate was increased in the first 50 °C. External factors of a volatile matter, including higher air velocity, lower air humidity, and higher air temperature contribute to the increase in drying rate [47]. The drying curve represents the loss of volatiles first by evaporation from a saturated surface above the ink film followed in turn by a period of evaporation from a saturated surface of gradually decreasing area and finally when the volatile compounds evaporate from the interior of the ink’s solid [48]. Sample S35 recorded the highest percentage of weight loss at 64.1% followed by sample S19 (55.8%), and S26 (53.4%). It can be concluded that the sample S35 with the highest viscosity showed the fastest drying time.

**Figure 8:**
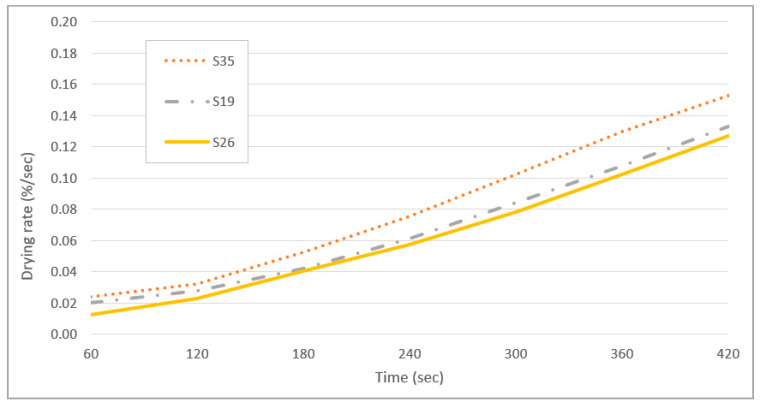
Weight loss as a function of time of sample S35, S19, and S26.

Figure 8 shows the drying rate curves as a function of time in seconds. The drying rates for samples S19 and S26 were comparable at between 0.01% and 0.13% per second. Sample S35 shows the fastest drying rate of approximately 0.15% per second over 420 s. Due to the low viscosity of sample S35, the diffusion of solvent molecules (water) proceeds at a faster rate than a higher viscosity sample which thus has a slower ability to evaporate [49].

The slower drying rate of highly viscous samples is due to diffusional mass transport limitations in the particle phase, arising from the viscous phase of the particles or due to a combination of oligomer degradation and mass transfer limitations [49]. If a viscous phase is formed, the mixing within the particle bulk will be kinetically limited, and an equilibrium process cannot represent the gas-particle partitioning. The drying rate is mainly governed by the reversible decomposition of oligomers back to monomers [50].

## 4. Conclusions

For the color lightness test, all factors and interaction effects were highly significant (*p* < 0.000) except for CMC. For the viscosity test, all factors and their interactions were highly significant (*p* < 0.000) except for glycerol. The contour plot indicated that the lowest amount of glycerol results in the lowest value of color lightness. A low amount of CMC results in low viscosity. The optimum formulation of marker ink can be achieved when the ratio of glycerol, benzalkonium chloride, ferrous sulphate, and CMC is set at 5, 5, 1, and 3, respectively. SEM micrographs showed the largest amount of cell wall structure collapse in leaf samples boiled with the lowest amount of glycerol as compared to other samples boiled in the lower amount of glycerol. Sample S35 recorded the highest percentage of weight loss at 64.1% followed by sample S19 (55.8%) and S26 (53.4%). It can be concluded that the sample S35, which had the highest viscosity, showed the fastest drying time. Several potential studies have been identified for future investigation including color saturation, anthocyanin content, and color components.

## Figures and Tables

**Table 1:** Factors and levels.

Factor	Unit	Notation	Levels				
			−2	−1	0	1	2
CMC	wt %	V_1_	1	2	3	4	5
Glycerol	wt %	V_2_	5	10	15	20	25

**Table 2:** Total number of experimental runs for full factorial design and RSM based on five-level factors

Factors	Levels	Total Number of Experimental Runs	
		Full Factorial Design	RSM
2	5	25	14
3	5	125	20
4	5	625	30
5	5	3125	54

**Table 3:** Design matrix.

Sample	Coded Factor		Uncoded Factor	
	V_1_	V_2_	V_1_	V_2_
S1	−2	0	5	3
S2	0	2	15	5
S3	1	−1	20	2
S4	−1	1	10	4
S5	−2	0	5	3
S6	0	0	15	3
S7	−1	−1	10	2
S8	0	0	15	3
S9	2	0	25	3
S10	0	0	15	3
S11	0	0	15	3
S12	0	−2	15	1
S13	1	−1	20	2
S14	0	0	15	3
S15	−2	0	5	3
S16	−1	−1	10	2
S17	−1	−1	10	2
S18	0	0	15	3
S19	1	1	20	4
S20	0	0	15	3
S21	0	0	15	3
S22	0	0	15	3
S23	1	−1	20	2
S24	1	1	20	4
S25	0	0	15	3
S26	−1	1	10	4
S27	0	−2	15	1
S28	0	2	15	5
S29	0	0	15	3
S30	0	0	15	3
S31	2	0	25	3
S32	0	−2	15	1
S33	−1	1	10	4
S34	0	0	15	3
S35	2	0	25	3
S36	1	1	20	4
S37	0	0	15	3
S38	0	2	15	5
S39	0	0	15	3

**Table 4:** Design matrix and response value for the viscosity and color lightness test.

Sample	Glycerol (V_1_)	CMC (V_2_)	Glycerol (V_1_)	CMC (V_2_)	Viscosity (Pa.s)	Color lightness (L*)
S1	−2	0	5	3	0.0221	24.70
S2	0	2	15	5	0.7278	25.83
S3	1	−1	20	2	0.0132	27.20
S4	−1	1	10	4	0.2320	25.11
S5	−2	0	5	3	0.0163	24.73
S6	0	0	15	3	0.1137	25.79
S7	−1	−1	10	2	0.0076	24.93
S8	0	0	15	3	0.1090	25.78
S9	2	0	25	3	0.0091	28.80
S10	0	0	15	3	0.1117	25.62
S11	0	0	15	3	0.1098	25.58
S12	0	−2	15	1	0.0045	25.83
S13	1	−1	20	2	0.0111	27.16
S14	0	0	15	3	0.1083	25.80
S15	−2	0	5	3	0.0203	24.78
S16	−1	−1	10	2	0.0109	25.14
S17	−1	−1	10	2	0.0084	24.99
S18	0	0	15	3	0.1042	25.61
S19	1	1	20	4	0.1595	27.40
S20	0	0	15	3	0.0948	25.78
S21	0	0	15	3	0.1077	25.84
S22	0	0	15	3	0.1064	25.85
S23	1	−1	20	2	0.0211	27.13
S24	1	1	20	4	0.1571	27.48
S25	0	0	15	3	0.1063	25.74
S26	−1	1	10	4	0.2245	25.09
S27	0	−2	15	1	0.0045	25.70
S28	0	2	15	5	0.7082	25.85
S29	0	0	15	3	0.1037	25.63
S30	0	0	15	3	0.1083	25.72
S31	2	0	25	3	0.0096	28.78
S32	0	−2	15	1	0.0045	25.62
S33	−1	1	10	4	0.2173	25.22
S34	0	0	15	3	0.0973	25.72
S35	2	0	25	3	0.0100	28.78
S36	1	1	20	4	0.1344	27.00
S37	0	0	15	3	0.0960	25.73
S38	0	2	15	5	0.6262	25.72
S39	0	0	15	3	0.1083	25.76

**Table 5:** Estimated effects and coefficient for glycerol and CMC on the color lightness.

Term	Notation	Coefficient	Std. Error of Coefficient	*p*
Constant		25.7739	0.03071	0.000
Glycerol	V_1_	1.0331	0.02135	0.000
CMC	V_2_	0.0347	0.02135	0.113
Glycerol*Glycerol	V_1_* V_1_	0.2607	0.01545	0.000
CMC*CMC	V_2_* V_2_	0.0098	0.01545	0.529
R^2^ = 0.9873 R^2^ (adj) = 0.9858				

**Table 6:** Estimated effects and coefficient for glycerol and CMC on the viscosity test

Term	Notation	Coefficient	Std. Error of Coefficient	*p*
Constant		0.0938	0.01191	0.000
Glycerol	V_1_	−0.0073	0.00828	0.382
CMC	V_2_	0.1431	0.00828	0.000
Glycerol*Glycerol	V_1_* V_1_	−0.0235	0.00599	0.000
CMC*CMC	V_2_* V_2_	0.0593	0.00599	0.000
R^2^ = 0.9292 R^2^ (adj) = 0.9209				

**Table 7:** Experimental validation for mangosteen-leaves-based marker ink properties.

Sample	Color Lightness (L*)			Viscosity (Pa.s)		
	Experimental Value	Predicted Value	Error (%)	Experimental Value	Predicted Value	Error (%)
SV1	24.99	24.75	0.97	0.0144	0.0143	0.70
SV2	24.80	24.75	0.20	0.0152	0.0143	6.29
SV3	24.83	24.75	0.32	0.0172	0.0143	20.28
	$\bar{x}$ Error		0.50	$\bar{x}$ Error		9.09

## Data Availability

The data presented in this study are available on request from the corresponding author.
